# Emerging ethnic differences in lung cancer therapy

**DOI:** 10.1038/sj.bjc.6604721

**Published:** 2008-11-04

**Authors:** I Sekine, N Yamamoto, K Nishio, N Saijo

**Affiliations:** 1 Division of Internal Medicine and Thoracic Oncology, National Cancer Center Hospital, Tsukiji 5-1-1, Chuo-ku, Tokyo 104-0045, Japan; 2 Department of Genome Biology, Kinki University School of Medicine, Sayama 589-8511, Japan; 3 Division of Internal Medicine, National Cancer Center Hospital East, Kashiwanoha 6-5-1, Kashiwa 277-8577, Japan

**Keywords:** lung cancer, ethnicity, epidermal growth factor receptor, pharmacogenomic

## Abstract

Although global clinical trials for lung cancer can enable the development of new agents efficiently, whether the results of clinical trials performed in one population can be fully extrapolated to another population remains questionable. A comparison of phase III trials for the same drug combinations against lung cancer in different countries shows a great diversity in haematological toxicity. One possible reason for this diversity may be that different ethnic populations may have different physiological capacities for white blood cell production and maturation. In addition, polymorphisms in the promoter and coding regions of drug-metabolising enzymes (e.g., CYP3A4 and UGT1A1) or in transporters (e.g., ABCB1) may vary among different ethnic populations. For example, epidermal growth factor receptor (EGFR) inhibitors are more effective in Asian patients than in patients of other ethnicities, a characteristic that parallels the incidence of EGFR-activating mutations. Interstitial lung disease associated with the administration of gefitinib is also more common among Japanese patients than among patients of other ethnicities. Although research into these differences has just begun, these studies suggest that possible pharmacogenomic and tumour genetic differences associated with individual responses to anticancer agents should be carefully considered when conducting global clinical trials.

Lung cancer is the most common malignancy worldwide. Approximately 1.2 million people are diagnosed with lung cancer annually (accounting for 12.3% of all cancers); the second most common malignancy is breast cancer (10.4%), followed by colorectal cancer (9.4%). As lung cancer almost invariably has a poor prognosis, it is the largest single cause of death from cancer in the world, with a mortality of 1.1 million annually (Stewart and Kleihues, 2003). Only 15% of lung cancer patients have a disease that is confined to the lung and are candidates for surgical resection; most patients with this disease have distant metastases or pleural effusion at the time of their initial diagnosis. These patients can be treated with systemic chemotherapy, but the efficacy of currently available anticancer agents is limited and patients with advanced diseases rarely live long.

As the development of new anticancer agents and chemotherapeutic regimens is both time and money consuming, clinical trials need to be as efficient as possible. One effort in this direction has been the adoption of global clinical trials for new agents that involve trial centres on more than one continent; this strategy enables adequate sample sizes to be obtained in a relatively short-time period and eliminates the need for redundant clinical trials with similar objectives conducted in different countries. However, whether the results of clinical trials performed in one population can be fully extrapolated to other populations remains questionable because of potential differences in trial designs, study-specific criteria, patient demographics, frequency of monitoring, and population-related pharmacokinetics, pharmacodynamics and pharmacogenomics. Recently, these genetic and physiologic factors influencing cancer chemotherapy have been increasingly examined and reported.

## Clinical observations of toxicity during cytotoxic chemotherapy

A comparison of phase III trials for the same drug combinations against non-small cell lung cancer conducted in different countries shows a great diversity in toxicity (Sekine *et al*, 2006). Among trials studying the combination of carboplatin and paclitaxel, the dose of carboplatin was fixed in all the trials, but the dose of paclitaxel was 200 mg m^−2^ in Japanese and European trials and 225 mg m^−2^ in American trials. Grades 3–4 neutropenia was noted in 88% of the patients in the Japanese trial, 15–51% of the patients in the European trials, and 6–65% of the patients in the American trials. Meanwhile, grades 3–4 febrile neutropenia was encountered in 16% of the patients in the Japanese trial, 0–9% of the patients in the European trials, and 2–4% of the patients in the American trials (Table 1). For combinations of cisplatin and docetaxel (Table 1) and cisplatin and vinorelbine (Table 2), the incidences of grades 3–4 neutropenia and febrile neutropenia were almost the same between phase III trials performed in different areas, but the doses of docetaxel and vinorelbine in the Japanese trials were lower than those in the European and American trials. Thus, neutropenia in patients receiving a combination of platinum and antimicrotubule agents may be more severe in Japanese than in Europeans and Americans. A higher frequency of grades 3–4 neutropenia in Japanese patients than in American patients was associated with combinations of cisplatin and irinotecan (65 *vs* 32%, *P*<0.001) and cisplatin and etoposide (92 *vs* 66%, *P*<0.001) for the treatment of extensive small-cell lung cancer (Lara *et al*, 2007).

How can this ethnic difference in the severity of neutropenia be explained? One possibility is that the physiological capacity of the white blood cell production and maturation may vary among different ethnic populations. An asymptomatic reduction in neutrophils (benign neutropenia) is more commonly observed in individuals of African descent than in Caucasians, and no data on this phenomenon are available for Asians (Hsieh *et al*, 2007). The mechanisms are unclear, but a lower bone marrow reserve, an intrinsic marrow difference, an abnormal cytokine response, or any combination of these factors have been suggested (Hsieh *et al*, 2007). The lower neutrophil counts were associated with higher levels of IL-8 and granulocyte colony-stimulating factor in African volunteers. Thus, these cytokines are considered to compensate for the relatively low neutrophil counts in this population (Mayr *et al*, 2007). A recent report showed that ethnicity-related low neutrophil counts were associated with neutrophil elastase (ELA2) polymorphisms (C-199A), but not with serum cytokine levels (Grann *et al*, 2007).

## Ethnic differences in drug metabolising enzymes

An explanation for the ethnic differences in haematological toxicity may be the varying activities of drug-metabolising enzymes and transporters that are mainly associated with polymorphisms in the promoter and coding regions of these enzymes (Fujita and Sasaki, 2007). The haematological toxicity of docetaxel monotherapy was associated with the clearance of this agent in Asian patients, a phenomenon that can be largely explained by CYP3A4 activity (Yamamoto *et al*, 2000). A study conducted in the Netherlands showed that docetaxel clearance was associated with the homozygous C1236T polymorphism in the ABCB1 (p-glycoprotein) gene (ABCB1*8) but was not associated with any CYP3A4 gene polymorphisms (Bosch *et al*, 2006). In contrast, docetaxel pharmacokinetics were not associated with the percent decrease in neutrophil counts nor with any polymorphisms in the CYP3A4 and ABCB1 genes in American patients (Lewis *et al*, 2007). Another example of ethnic differences in drug-metabolising enzymes is the association between polymorphisms in genes involved in irinotecan metabolism and irinotecan-induced neutropenia. Among the patients who received irinotecan with or without another anticancer agent, grade 4 neutropenia was noted in 40–57% of the patients with UDP-glucuronosyltransferase (UGT) 1A1^*^28 (a polymorphism in the promoter region of the UGT1A1 gene) homozygosity, whereas neutropenia was only observed in 15% or less of the patients with wild-type alleles. This association was consistent in both Asian and Caucasian patients, although the frequency of homozygosity was about 10% in Caucasians and much lower in Asians. The UGT1A1*6 allele is another polymorphism at exon 1 that is associated with defective glucuronidating function and is found almost exclusively in Asian individuals with a frequency as high as 20% (Fujita and Sasaki, 2007). UGT1A1*6 is significantly linked to polymorphisms of UGT1A7 and UGT1A9. A haplotype including UGT1A1*6 and UGT1A7*3, noted in as many as 15% of Japanese patients, and UGT1A1*6 homozygosity, noted in 7% of Korean patients, were significantly associated with decreased glucuronosyltransferase activity for SN-38 and severe neutropenia (Han *et al*, 2006; Fujita *et al*, 2007). In 177 Japanese patients treated with irinotecan-including chemotherapy, a homozygous or double heterozygous genotype for UGT1A1^*^6 and UGT1A1^*^28 (^*^6/^*^6, ^*^28/^*^28 or ^*^6/^*^28) was significantly associated with severe neutropenia (Minami *et al*, 2007). In addition, patients with a homozygous C3435T polymorphism in the ABCB1 gene are four-fold more likely to develop grade 3 diarrhoea when treated with a combination of cisplatin and irinotecan (Lara *et al*, 2007).

Data on associations between polymorphisms in genes coding drug-metabolising enzymes and therapeutic efficacy remain scarce. A recent prospective study in 250 patients with metastatic colorectal cancer showed a significantly higher response rate (67 *vs* 40%) and a nonsignificant survival advantage (hazard ratio (HR): 0.81; 95% confidence interval (CI): 0.45–1.44) in patients homozygous for UGT1A1^*^28, compared with those with wild-type alleles; these outcomes were associated with a higher exposure to SN-38 (Toffoli *et al*, 2006). In a study of 81 NSCLC patients, those who were homozygous for UGT1A1^*^6 had a lower response rate (0 *vs* 50%, *P*=0.038) and a poorer MST (7.6 *vs* 17.7 months, *P*=0.017) as well as greater toxicities than the other patients (Han *et al*, 2006). The most plausible explanation for the negative effects of UGT1A1^*^6 on treatment outcome may be that the dose intensity or cycle number might have been reduced in patients with UGT1A1^*^6 because of polymorphism-associated toxicities (Fujita and Sasaki, 2007).

These pharmacogenetic analyses have been rather preliminary. Data on genotyping, pharmacokinetics, and pharmacodynamics collected from a large number of patients with different ethnic backgrounds are needed to demonstrate the cause of ethnic differences in chemotherapy-associated toxicity.

## Efficacy of epidermal growth factor receptor tyrosine kinase inhibitors

Epidermal growth factor receptor (EGFR), a cell membrane receptor with tyrosine kinase activity, is expressed in most patients with NSCLC and plays a role in cellular proliferation, inhibition of apoptosis, angiogenesis, metastatic potential, and chemoresistance. Small-molecule inhibitors of EGFR, such as gefitinib and erlotinib, have shown antitumor activity and have alleviated symptoms in NSCLC patients who were previously treated with standard chemotherapy. Two randomized phase II studies, IDEAL (Iressa Dose Evaluation in Advanced Lung Cancer)-1 (involving 210 patients and conducted in Europe, Australia, South Africa, and Japan) and IDEAL-2 (involving 216 patients and conducted in the USA), have evaluated the efficacy of gefitinib at a dose of either 250 mg daily or 500 mg daily in patients with advanced NSCLC in whom earlier platinum-based chemotherapy had failed. No difference in the response rates between the doses was noted, but an increased response rate was recorded for never smokers, women, and those with an adenocarcinoma histology, compared with patients who did not have these characteristics. In addition, the response rate was 28% in Japanese patients but only 9–12% in patients of other ethnicities (Fukuoka *et al*, 2003; Kris *et al*, 2003). A randomized phase III trial, ISEL (Iressa Survival Evaluation in Lung Cancer), of gefitinib *vs* a placebo in 1692 NSCLC patients who had been previously treated with one or two chemotherapeutic regimens failed to show any survival benefit of gefitinib; in the overall population, the median survival times (MSTs) in the gefitinib and placebo arms were 5.6 and 5.1 months, respectively (HR: 0.89; 95% CI: 0.78–1.03). A subgroup analysis, however, showed that the MST was longer in Asian patients receiving gefitinib than in those receiving the placebo (MST: 9.5 *vs* 5.5 months; HR: 0.66; 95% CI: 0.48–0.91). Similar results were seen for never smokers: patients receiving gefitinib survived longer than those receiving the placebo (MST: 8.9 *vs* 6.1 months; HR: 0.67, 95% CI: 0.49–0.91) (Thatcher *et al*, 2005).

A similar association between objective responses and ethnicity was observed in studies on erlotinib monotherapy for previously treated advanced NSCLC. In an American phase II trial of this agent in 57 advanced NSCLC patients with disease progression or relapse after platinum-based chemotherapy, the response rate was 12% and the MST was 8.4 months (Perez-Soler *et al*, 2004). In contrast, the combined data of two Japanese phase II trials of erlotinib in similar patient populations showed objective responses in 30 of 106 (28%) patients and an MST of 13.8 months. Among the responders, significantly higher proportions of females (50%) than males (17%) (*P*=0.0009) and of never smokers (51%) than smokers (14%) were observed (*P*<0.0001) (Tamura *et al*, 2007). A phase III trial of erlotinib or a placebo in 731 NSCLC patients previously treated with one or two chemotherapy regimens showed that the response rate in Asian patients was higher than that in patients of other ethnicities (28 *vs* 10%, *P*=0.02) (Shepherd *et al*, 2005).

These results of phases II and III trials consistently suggest that EGFR tyrosine kinase inhibitors may be more effective in Asian patients than in patients of other ethnicities.

In April 2004, the activating mutations of the EGFR gene were identified in NSCLC specimens, and cancers with these mutations were reported to be highly sensitive to gefitinib. The populations with higher responses to gefitinib (females, non-smokers and patients with an adenocarcinoma histology) also have higher incidences of EGFR mutations (Kosaka *et al*, 2004; Pao *et al*, 2004; Shigematsu *et al*, 2005). The incidence of EGFR mutations in surgically resected tissue samples is summarised in Table 3 (Kosaka *et al*, 2004; Pao *et al*, 2004; Marchetti *et al*, 2005; Qin *et al*, 2005; Shigematsu *et al*, 2005; Soung *et al*, 2005; Tokumo *et al*, 2005; Yang *et al*, 2005; Sasaki *et al*, 2006). The incidence varies from one report to another, but EGFR mutations tend to be more common among patients with an adenocarcinoma histology and among non-smokers. Among Asian patients, the average incidences of EGFR mutations were 31% overall, 47% among patients with adenocarcinoma, and 56% among non-smokers; among other ethnic populations, however, the average incidences were 7–8% overall, 13–15% among patients with adenocarcinoma, and 34–35% among non-smokers (Table 3). Thus, the percentage of responders to gefitinib or erlotinib almost paralleled the percentage of patients with EGFR mutations.

The mechanism responsible for the high frequency of EGFR mutations in Asian patients is a subject of great interest, and polymorphisms in the regulatory sequence of the EGFR gene have been vigorously investigated. The CA simple sequence repeat 1 (CA-SSR1), a highly polymorphic locus containing 14–21 CA dinucleotide repeats, is located at the 5′ end of intron 1 of the EGFR gene. Studies of CA-SSR1 repeat length and EGFR expression in breast cancer tissues have shown a constant decline in EGFR expression with increasing repeat length (Buerger *et al*, 2000, 2004). In addition, a shorter repeat length was associated with an elevated risk of lung cancer (Zhang *et al*, 2007) and poor survival in NSCLC patients (Dubey *et al*, 2006). The CA-SSR1 repeat length distribution varies according to ethnicity, with Asians tending to have longer repeats than Americans (Liu *et al*, 2003). Two single-nucleotide polymorphisms in the promoter region of the EGFR gene (−219G/T and −191C/A) were also associated with promoter activity and EGFR expression (Liu *et al*, 2005), and their polymorphic types (associated with low EGFR expression) were more common among Asians than among other ethnicities (Nomura *et al*, 2007). These observations suggest that many Asians have polymorphic types that lead to a decreased intrinsic production of EGFR protein. If a certain critical level of EGFR is required to drive the cell toward a malignant phenotype, another mechanism including activating mutations of EGFR and/or the autonomous activation of downstream signalling may be required for the development of lung cancer among Asians (Nomura *et al*, 2007).

## Interstitial lung disease associated with gefitinib and erlotinib

The frequencies of grades 3–4 common toxicities after the administration of gefitinib, including diarrhoea, skin rash, and elevated liver transaminase levels, have been similar among study populations, but the incidence of severe interstitial lung disease (ILD) associated with the administration of gefitinib differs between patients in Japan and those in other countries. In the IDEAL studies, two Japanese patients developed grades 3–4 ILD (2%), whereas no patients outside of Japan experienced ILD (Fukuoka *et al*, 2003; Kris *et al*, 2003). A retrospective study of 1976 consecutive patients treated with gefitinib at 84 institutions showed that the incidence of ILD was 3.5% and the mortality rate was 1.6%. Several risk factors for the development of gefitinib-induced ILD were identified in the Japanese population: a history of pulmonary fibrosis, a history of smoking, a poor performance status, and a male sex (Ando *et al*, 2006). A similar incidence of ILD (4.6%) was also noted in association with erlotinib chemotherapy in Japanese phase II trials (Tamura *et al*, 2007).

The association between ILD and anticancer treatment is a major topic in Japan because (1) the diagnosis of ILD can be difficult and a consensus among physicians is sometimes not reached, (2) the risk factors for ILD have not been fully established, 3) an effective treatment for ILD has not been established and the condition is often fatal, and (4) the low frequency of this complication makes it difficult to conduct pertinent clinical trials. Gefitinib-induced ILD seems to be more common among Japanese patients than among other patients, but the reasons for this ethnic difference are totally unknown.

## Conclusion

The findings discussed here suggest that considerable variations in the toxicity and efficacy of anticancer agents may exist among patients of different ethnicities. Although research into these differences has just begun, these studies suggest that possible pharmacogenomic and tumour genetic differences associated with individual responses to anticancer agents should be carefully considered when conducting global clinical trials.

## Figures and Tables

**Table 1:** Toxicity associated with a combination of platinum and taxane

	**Chemotherapy dose**			**Grades 3–4 toxicity (%)**		
**Research group**	**Platinum**	**Taxane**	**No. of patients**	**NP**	**FNP**	**Reference**
*A combination of carboplatin and paclitaxel*						
Japan	6 (AUC)	200 (mg m^−2^)	145	88	16	Ohe *et al* (2007)
Greece	6 (AUC)	200 (mg m^−2^)	252	15	0	Kosmidis *et al* (2002)
EU	6 (AUC)	200 (mg m^−2^)	309	51	4	Rosell *et al* (2002)
ECOG	6 (AUC)	225 (mg m^−2^)	290	63	4	Schiller *et al* (2002)
SWOG	6 (AUC)	225 (mg m^−2^)	206	57	2	Kelly *et al* (2001)
SWOG	6 (AUC)	225 (mg m^−2^)	182	—	3	Gandara *et al* (2004)
USA	6 (AUC)	225 (mg m^−2^)	190	65	—	Belani *et al* (2005)
USA	6 (AUC)	225 (mg m^−2^)	345	6	—	Herbst *et al* (2004)
						
*A combination of cisplatin and docetaxel*						
Japan	80 (mg m^−2^)	60 (mg m^−2^)	151	74	2	Ohe *et al* (2007)
ECOG	75 (mg m^−2^)	75 (mg m^−2^)	289	69	11	Schiller *et al* (2002)
USA	75 (mg m^−2^)	75 (mg m^−2^)	408	75	5	Fossella *et al* (2003)

NP, neutropenia; FNP, febrile neutropenia.

**Table 2:** Toxicity associated with a combination of cisplatin and vinorelbine

	**Chemotherapy dose (mg m**^−**2**^)			**Grades 3–4 toxicity (%)**		
**Research group**	**Cisplatin**	**Vinorelbine**	**No. of patients**	**NP**	**FNP**	**Reference**
Japan	80 (day 1)	25 (days 1, 8)	145	88	18	Ohe *et al* (2007)
Greece	80 (day 8)	30 (days 1, 8)	204	37	11	Georgoulias *et al* (2005)
France	100 (day 1)	30 (weekly)	156	83	22	Pujol *et al* (2005)
EU	120 (day 1)	30 (weekly)	206	79	4	Le Chevalier *et al* (1994)
SWOG	100 (day 1)	25 (weekly)	202	76	1	Kelly *et al* (2001)
USA	100 (day 1)	25 (weekly)	404	79	5	Fossella *et al* (2003)

NP, neutropenia; FNP, febrile neutropenia.

**Table 3:** Incidence of EGFR mutations in surgically resected specimens

		**All cases**		**Adenocarcinoma**		**Non-smokers**	
		**Total**	**Mutation**	**Total**	**Mutation**	**Total**	**Mutation**
**Author**	**Country**	***N***	***N* (%)**	***N***	***N* (%)**	***N***	***N* (%)**
*Western areas*							
Shigematsu	USA	80	11 (14)	44	11 (25)	26	7 (27)
Pao	USA	96	11 (11)	72	11 (15)	15	7 (47)
Yang	USA	219	26 (12)	164	25 (15)	34	12 (35)
Marchetti	Italy	860	39 (5)	375	39 (10)	103^a^	23 (22)
							
	Subtotal	1255	87 (7)	655	86 (13)	75	26 (35)
							
*Asian areas*							
Shigematsu	Japan	263	71 (27)	154	67 (44)	78	47 (60)
Kosaka	Japan	277	111 (40)	224	110 (49)	112^a^	76 (68)
Tokumo	Japan	120	38 (32)	82	37 (45)	36	25 (69)
Sasaki	Japan	95	35 (37)	71	32 (45)	36	25 (69)
Shigematsu	Taiwan	93	32 (34)	55	31 (56)	55	27 (49)
Qin	China	41	10 (24)	17	7 (41)	21	6 (29)
Soung	Korea	153	30 (20)	69	26 (38)	54	25 (46)
Shigematsu	Others	361	107 (30)	214	102 (48)	135	76 (56)
							
	Subtotal	1403	434 (31)	886	412 (47)	415	231 (56)
							
*Other areas*							
Shigematsu	Australia	83	6 (7)	36	5 (14)	7	4 (57)
Shigematsu	Others	158	13 (8)	75	12 (16)	31	9 (29)
							
	Subtotal	241	19 (8)	111	17 (15)	38	13 (34)
							
	Toatl	2899	540 (19)	1652	515 (31)	528	270 (51)

a Including only patients with adenocarcinoma histology.

## References

[bib1] Ando M, Okamoto I, Yamamoto N, Takeda K, Tamura K, Seto T, Ariyoshi Y, Fukuoka M (2006) Predictive factors for interstitial lung disease, antitumor response, and survival in non-small-cell lung cancer patients treated with gefitinib. J Clin Oncol 24: 2549–2556 16735708 10.1200/JCO.2005.04.9866

[bib2] Belani CP, Lee JS, Socinski MA, Robert F, Waterhouse D, Rowland K, Ansari R, Lilenbaum R, Natale RB (2005) Randomized phase III trial comparing cisplatin-etoposide to carboplatin-paclitaxel in advanced or metastatic non-small cell lung cancer. Ann Oncol 16: 1069–1075 15860487 10.1093/annonc/mdi216

[bib3] Bosch TM, Huitema AD, Doodeman VD, Jansen R, Witteveen E, Smit WM, Jansen RL, van Herpen CM, Soesan M, Beijnen JH, Schellens JH (2006) Pharmacogenetic screening of CYP3A and ABCB1 in relation to population pharmacokinetics of docetaxel. Clin Cancer Res 12: 5786–5793 17020985 10.1158/1078-0432.CCR-05-2649

[bib4] Buerger H, Gebhardt F, Schmidt H, Beckmann A, Hutmacher K, Simon R, Lelle R, Boecker W, Brandt B (2000) Length and loss of heterozygosity of an intron 1 polymorphic sequence of egfr is related to cytogenetic alterations and epithelial growth factor receptor expression. Cancer Res 60: 854–857 10706093

[bib5] Buerger H, Packeisen J, Boecker A, Tidow N, Kersting C, Bielawski K, Isola J, Yatabe Y, Nakachi K, Boecker W, Brandt B (2004) Allelic length of a CA dinucleotide repeat in the egfr gene correlates with the frequency of amplifications of this sequence – first results of an inter-ethnic breast cancer study. J Pathol 203: 545–550 15095477 10.1002/path.1542

[bib6] Dubey S, Stephenson P, Levy DE, Miller JA, Keller SM, Schiller JH, Johnson DH, Kolesar JM (2006) EGFR dinucleotide repeat polymorphism as a prognostic indicator in non-small cell lung cancer. J Thorac Oncol 1: 406–412 17409891

[bib7] Fossella F, Pereira JR, von Pawel J, Pluzanska A, Gorbounova V, Kaukel E, Mattson KV, Ramlau R, Szczesna A, Fidias P, Millward M, Belani CP (2003) Randomized, multinational, phase III study of docetaxel plus platinum combinations versus vinorelbine plus cisplatin for advanced non-small-cell lung cancer: the TAX 326 study group. J Clin Oncol 21: 3016–3024 12837811 10.1200/JCO.2003.12.046

[bib8] Fujita K, Ando Y, Nagashima F, Yamamoto W, Eodo H, Araki K, Kodama K, Miya T, Narabayashi M, Sasaki Y (2007) Genetic linkage of UGT1A7 and UGT1A9 polymorphisms to UGT1A1^*^6 is associated with reduced activity for SN-38 in Japanese patients with cancer. Cancer Chemother Pharmacol 60: 515–522 17406868 10.1007/s00280-006-0396-1

[bib9] Fujita K, Sasaki Y (2007) Pharmacogenomics in drug-metabolizing enzymes catalyzing anticancer drugs for personalized cancer chemotherapy. Curr Drug Metab 8: 554–562 17691917 10.2174/138920007781368890

[bib10] Fukuoka M, Yano S, Giaccone G, Tamura T, Nakagawa K, Douillard JY, Nishiwaki Y, Vansteenkiste J, Kudoh S, Rischin D, Eek R, Horai T, Noda K, Takata I, Smit E, Averbuch S, Macleod A, Feyereislova A, Dong RP, Baselga J (2003) Multi-institutional randomized phase II trial of gefitinib for previously treated patients with advanced non-small-cell lung cancer (The IDEAL 1 Trial) [corrected]. J Clin Oncol 21: 2237–2246 12748244 10.1200/JCO.2003.10.038

[bib11] Gandara DR, Ohe Y, Kubota K, Nishiwaki Y, Ariyoshi Y, Saijo N, Williamson S, Lara PN, Crowley J, Fukuoka M (2004) Japan-SWOG common arm analysis of paclitaxel/carboplatin in advanced stage non-small cell lung cancer (NSCLC): a model for prospective comparison of cooperative group trials. Proc ASCO 22: 618s

[bib12] Georgoulias V, Ardavanis A, Tsiafaki X, Agelidou A, Mixalopoulou P, Anagnostopoulou O, Ziotopoulos P, Toubis M, Syrigos K, Samaras N, Polyzos A, Christou A, Kakolyris S, Kouroussis C, Androulakis N, Samonis G, Chatzidaki D (2005) Vinorelbine plus cisplatin versus docetaxel plus gemcitabine in advanced non-small-cell lung cancer: a phase III randomized trial. J Clin Oncol 23: 2937–2945 15728228 10.1200/JCO.2005.04.016

[bib13] Grann V, Bowman N, Wei Y, Horwitz M, Joseph C, Abdul K, Abdul K, Barlatier H, Sandoval R, Jacobson J, Hershman D (2007) Ethnic neutropenia among women of European, African, and Caribbean backgrounds. J Clin Oncol 25(Suppl): 343s (abstract 6587)

[bib14] Han JY, Lim HS, Shin ES, Yoo YK, Park YH, Lee JE, Jang IJ, Lee DH, Lee JS (2006) Comprehensive analysis of UGT1A polymorphisms predictive for pharmacokinetics and treatment outcome in patients with non-small-cell lung cancer treated with irinotecan and cisplatin. J Clin Oncol 24: 2237–2244 16636344 10.1200/JCO.2005.03.0239

[bib15] Herbst RS, Giaccone G, Schiller JH, Natale RB, Miller V, Manegold C, Scagliotti G, Rosell R, Oliff I, Reeves JA, Wolf MK, Krebs AD, Averbuch SD, Ochs JS, Grous J, Fandi A, Johnson DH (2004) Gefitinib in combination with paclitaxel and carboplatin in advanced non-small-cell lung cancer: a phase III trial – INTACT 2. J Clin Oncol 22: 785–794 14990633 10.1200/JCO.2004.07.215

[bib16] Hsieh MM, Everhart JE, Byrd-Holt DD, Tisdale JF, Rodgers GP (2007) Prevalence of neutropenia in the US population: age, sex, smoking status, and ethnic differences. Ann Intern Med 146: 486–492 17404350 10.7326/0003-4819-146-7-200704030-00004

[bib17] Kelly K, Crowley J, Bunn Jr PA, Presant CA, Grevstad PK, Moinpour CM, Ramsey SD, Wozniak AJ, Weiss GR, Moore DF, Israel VK, Livingston RB, Gandara DR (2001) Randomized phase III trial of paclitaxel plus carboplatin versus vinorelbine plus cisplatin in the treatment of patients with advanced non-small-cell lung cancer: a Southwest Oncology Group trial. J Clin Oncol 19: 3210–3218 11432888 10.1200/JCO.2001.19.13.3210

[bib18] Kosaka T, Yatabe Y, Endoh H, Kuwano H, Takahashi T, Mitsudomi T (2004) Mutations of the epidermal growth factor receptor gene in lung cancer: biological and clinical implications. Cancer Res 64: 8919–8923 15604253 10.1158/0008-5472.CAN-04-2818

[bib19] Kosmidis P, Mylonakis N, Nicolaides C, Kalophonos C, Samantas E, Boukovinas J, Fountzilas G, Skarlos D, Economopoulos T, Tsavdaridis D, Papakostas P, Bacoyiannis C, Dimopoulos M (2002) Paclitaxel plus carboplatin versus gemcitabine plus paclitaxel in advanced non-small-cell lung cancer: a phase III randomized trial. J Clin Oncol 20: 3578–3585 12202657 10.1200/JCO.2002.12.112

[bib20] Kris MG, Natale RB, Herbst RS, Lynch Jr TJ, Prager D, Belani CP, Schiller JH, Kelly K, Spiridonidis H, Sandler A, Albain KS, Cella D, Wolf MK, Averbuch SD, Ochs JJ, Kay AC (2003) Efficacy of gefitinib, an inhibitor of the epidermal growth factor receptor tyrosine kinase, in symptomatic patients with non-small cell lung cancer: a randomized trial. JAMA 290: 2149–2158 14570950 10.1001/jama.290.16.2149

[bib21] Lara P, Redman M, Lenz H, Gordon M, Shibata T, Fukuda H, Tamura T, Saijo N, Natale R, Gandara D (2007) Cisplatin (Cis)/etoposide (VP16) compared to cis/irinotecan (CPT11) in extensive-stage small cell lung cancer (E-SCLC): pharmacogenomic (PG) and comparative toxicity analysis of JCOG 9511 and SWOG 0124. J Clin Oncol 25(Suppl): 390s (abstract 7524)

[bib22] Le Chevalier T, Brisgand D, Douillard JY, Pujol JL, Alberola V, Monnier A, Riviere A, Lianes P, Chomy P, Cigolari S, Gottfried M, Ruffle P, Panizo A, Gaspard MH, Ravaioli A, Besenval M, Besson F, Martinez A, Berthaud P, Tursz T (1994) Randomized study of vinorelbine and cisplatin versus vindesine and cisplatin versus vinorelbine alone in advanced non-small-cell lung cancer: results of a European multicenter trial including 612 patients. J Clin Oncol 12: 360–367 8113844 10.1200/JCO.1994.12.2.360

[bib23] Lewis LD, Miller AA, Rosner GL, Dowell JE, Valdivieso M, Relling MV, Egorin MJ, Bies RR, Hollis DR, Levine EG, Otterson GA, Millard F, Ratain MJ (2007) A comparison of the pharmacokinetics and pharmacodynamics of docetaxel between African-American and Caucasian cancer patients: CALGB 9871. Clin Cancer Res 13: 3302–3311 17545536 10.1158/1078-0432.CCR-06-2345

[bib24] Liu W, Innocenti F, Chen P, Das S, Cook Jr EH, Ratain MJ (2003) Interethnic difference in the allelic distribution of human epidermal growth factor receptor intron 1 polymorphism. Clin Cancer Res 9: 1009–1012 12631599

[bib25] Liu W, Innocenti F, Wu MH, Desai AA, Dolan ME, Cook Jr EH, Ratain MJ (2005) A functional common polymorphism in a Sp1 recognition site of the epidermal growth factor receptor gene promoter. Cancer Res 65: 46–53 15665278

[bib26] Marchetti A, Martella C, Felicioni L, Barassi F, Salvatore S, Chella A, Camplese PP, Iarussi T, Mucilli F, Mezzetti A, Cuccurullo F, Sacco R, Buttitta F (2005) EGFR mutations in non-small-cell lung cancer: analysis of a large series of cases and development of a rapid and sensitive method for diagnostic screening with potential implications on pharmacologic treatment. J Clin Oncol 23: 857–865 15681531 10.1200/JCO.2005.08.043

[bib27] Mayr FB, Spiel AO, Leitner JM, Firbas C, Kliegel T, Jilma B (2007) Ethnic differences in plasma levels of interleukin-8 (IL-8) and granulocyte colony stimulating factor (G-CSF). Transl Res 149: 10–14 17196517 10.1016/j.trsl.2006.06.003

[bib28] Minami H, Sai K, Saeki M, Saito Y, Ozawa S, Suzuki K, Kaniwa N, Sawada J, Hamaguchi T, Yamamoto N, Shirao K, Yamada Y, Ohmatsu H, Kubota K, Yoshida T, Ohtsu A, Saijo N (2007) Irinotecan pharmacokinetics/pharmacodynamics and UGT1A genetic polymorphisms in Japanese: roles of UGT1A1^*^6 and ^*^28. Pharmacogenet Genomics 17: 497–504 17558305 10.1097/FPC.0b013e328014341f

[bib29] Nomura M, Shigematsu H, Li L, Suzuki M, Takahashi T, Estess P, Siegelman M, Feng Z, Kato H, Marchetti A, Shay JW, Spitz MR, Wistuba II, Minna JD, Gazdar AF (2007) Polymorphisms, mutations, and amplification of the EGFR gene in non-small cell lung cancers. PLoS Med 4: e125 17455987 10.1371/journal.pmed.0040125 PMC1876407

[bib30] Ohe Y, Ohashi Y, Kubota K, Tamura T, Nakagawa K, Negoro S, Nishiwaki Y, Saijo N, Ariyoshi Y, Fukuoka M (2007) Randomized phase III study of cisplatin plus irinotecan versus carboplatin plus paclitaxel, cisplatin plus gemcitabine, and cisplatin plus vinorelbine for advanced non-small-cell lung cancer: Four-Arm Cooperative Study in Japan. Ann Oncol 18: 317–323 17079694 10.1093/annonc/mdl377

[bib31] Pao W, Miller V, Zakowski M, Doherty J, Politi K, Sarkaria I, Singh B, Heelan R, Rusch V, Fulton L, Mardis E, Kupfer D, Wilson R, Kris M, Varmus H (2004) EGF receptor gene mutations are common in lung cancers from ‘never smokers’ and are associated with sensitivity of tumors to gefitinib and erlotinib. Proc Natl Acad Sci USA 101: 13306–13311 15329413 10.1073/pnas.0405220101 PMC516528

[bib32] Perez-Soler R, Chachoua A, Hammond LA, Rowinsky EK, Huberman M, Karp D, Rigas J, Clark GM, Santabarbara P, Bonomi P (2004) Determinants of tumor response and survival with erlotinib in patients with non-small-cell lung cancer. J Clin Oncol 22: 3238–3247 15310767 10.1200/JCO.2004.11.057

[bib33] Pujol JL, Breton JL, Gervais R, Rebattu P, Depierre A, Morere JF, Milleron B, Debieuvre D, Castera D, Souquet PJ, Moro-Sibilot D, Lemarie E, Kessler R, Janicot H, Braun D, Spaeth D, Quantin X, Clary C (2005) Gemcitabine-docetaxel versus cisplatin-vinorelbine in advanced or metastatic non-small-cell lung cancer: a phase III study addressing the case for cisplatin. Ann Oncol 16: 602–610 15741225 10.1093/annonc/mdi126

[bib34] Qin BM, Chen X, Zhu JD, Pei DQ (2005) Identification of EGFR kinase domain mutations among lung cancer patients in China: implication for targeted cancer therapy. Cell Res 15: 212–217 15780185 10.1038/sj.cr.7290289

[bib35] Rosell R, Gatzemeier U, Betticher DC, Keppler U, Macha HN, Pirker R, Berthet P, Breau JL, Lianes P, Nicholson M, Ardizzoni A, Chemaissani A, Bogaerts J, Gallant G (2002) Phase III randomised trial comparing paclitaxel/carboplatin with paclitaxel/cisplatin in patients with advanced non-small-cell lung cancer: a cooperative multinational trial. Ann Oncol 13: 1539–1549 12377641 10.1093/annonc/mdf332

[bib36] Sasaki H, Shimizu S, Endo K, Takada M, Kawahara M, Tanaka H, Matsumura A, Iuchi K, Haneda H, Suzuki E, Kobayashi Y, Yano M, Fujii Y (2006) EGFR and erbB2 mutation status in Japanese lung cancer patients. Int J Cancer 118: 180–184 16003726 10.1002/ijc.21301

[bib37] Schiller JH, Harrington D, Belani CP, Langer C, Sandler A, Krook J, Zhu J, Johnson DH (2002) Comparison of four chemotherapy regimens for advanced non-small-cell lung cancer. N Engl J Med 346: 92–98 11784875 10.1056/NEJMoa011954

[bib38] Sekine I, Nokihara H, Yamamoto N, Kunitoh H, Ohe Y, Saijo N, Tamura T (2006) Common arm analysis: one approach to develop the basis for global standardization in clinical trials of non-small cell lung cancer. Lung Cancer 53: 157–164 16781004 10.1016/j.lungcan.2006.05.007

[bib39] Shepherd FA, Rodrigues Pereira J, Ciuleanu T, Tan EH, Hirsh V, Thongprasert S, Campos D, Maoleekoonpiroj S, Smylie M, Martins R, van Kooten M, Dediu M, Findlay B, Tu D, Johnston D, Bezjak A, Clark G, Santabarbara P, Seymour L (2005) Erlotinib in previously treated non-small-cell lung cancer. N Engl J Med 353: 123–132 16014882 10.1056/NEJMoa050753

[bib40] Shigematsu H, Lin L, Takahashi T, Nomura M, Suzuki M, Wistuba II, Fong KM, Lee H, Toyooka S, Shimizu N, Fujisawa T, Feng Z, Roth JA, Herz J, Minna JD, Gazdar AF (2005) Clinical and biological features associated with epidermal growth factor receptor gene mutations in lung cancers. J Natl Cancer Inst 97: 339–346 15741570 10.1093/jnci/dji055

[bib41] Soung YH, Lee JW, Kim SY, Seo SH, Park WS, Nam SW, Song SY, Han JH, Park CK, Lee JY, Yoo NJ, Lee SH (2005) Mutational analysis of EGFR and K-RAS genes in lung adenocarcinomas. Virchows Arch 446: 483–488 15815931 10.1007/s00428-005-1254-y

[bib42] Stewart B, Kleihues P (2003) The global burden of cancer. In World Cancer Report. International Agency for Research on Cancer, Stewart B, Kleihues P (eds), pp 11–19. IARC Press: Lyon

[bib43] Tamura T, Nishiwaki Y, Watanabe K, Nakagawa K, Matsui K, Takahashi T, Segawa Y, Ichinose Y, Fukuoka M, Saijo N (2007) Evaluation of efficacy and safety of erlotinib as monotherapy for Japanese patients with advanced non-small cell lung cancer (NSCLC); integrated analysis of two Japanese phase II studies. J Thorac Oncol 2(Suppl): s742

[bib44] Thatcher N, Chang A, Parikh P, Rodrigues Pereira J, Ciuleanu T, von Pawel J, Thongprasert S, Tan EH, Pemberton K, Archer V, Carroll K (2005) Gefitinib plus best supportive care in previously treated patients with refractory advanced non-small-cell lung cancer: results from a randomised, placebo-controlled, multicentre study (Iressa Survival Evaluation in Lung Cancer). Lancet 366: 1527–1537 16257339 10.1016/S0140-6736(05)67625-8

[bib45] Toffoli G, Cecchin E, Corona G, Russo A, Buonadonna A, D′Andrea M, Pasetto LM, Pessa S, Errante D, De Pangher V, Giusto M, Medici M, Gaion F, Sandri P, Galligioni E, Bonura S, Boccalon M, Biason P, Frustaci S (2006) The role of UGT1A1^*^28 polymorphism in the pharmacodynamics and pharmacokinetics of irinotecan in patients with metastatic colorectal cancer. J Clin Oncol 24: 3061–3068 16809730 10.1200/JCO.2005.05.5400

[bib46] Tokumo M, Toyooka S, Kiura K, Shigematsu H, Tomii K, Aoe M, Ichimura K, Tsuda T, Yano M, Tsukuda K, Tabata M, Ueoka H, Tanimoto M, Date H, Gazdar AF, Shimizu N (2005) The relationship between epidermal growth factor receptor mutations and clinicopathologic features in non-small cell lung cancers. Clin Cancer Res 11: 1167–1173 15709185

[bib47] Yamamoto N, Tamura T, Kamiya Y, Sekine I, Kunitoh H, Saijo N (2000) Correlation between docetaxel clearance and estimated cytochrome P450 activity by urinary metabolite of exogenous cortisol. J Clin Oncol 18: 2301–2308 10829051 10.1200/JCO.2000.18.11.2301

[bib48] Yang SH, Mechanic LE, Yang P, Landi MT, Bowman ED, Wampfler J, Meerzaman D, Hong KM, Mann F, Dracheva T, Fukuoka J, Travis W, Caporaso NE, Harris CC, Jen J (2005) Mutations in the tyrosine kinase domain of the epidermal growth factor receptor in non-small cell lung cancer. Clin Cancer Res 11: 2106–2110 15788655 10.1158/1078-0432.CCR-04-1853

[bib49] Zhang W, Weissfeld JL, Romkes M, Land SR, Grandis JR, Siegfried JM (2007) Association of the EGFR intron 1 CA repeat length with lung cancer risk. Mol Carcinog 46: 372–380 17219440 10.1002/mc.20285

